# Elimination of Over-the-Counter Medicines from the Health Insurance Coverage: A Right or Wrong Decision

**Published:** 2019-08

**Authors:** Hassan JOULAEI, Mohammadreza HEYDARI

**Affiliations:** Shiraz HIV/AIDS Research Center, Institute of Health, Shiraz University of Medical Sciences, Shiraz, Iran

## Dear Editor-in-Chief

Due to resource constraints, governments have to prioritize their funding to provide equitable health services for all people based on their needs rather than their ability to pay. One of the most common ways of doing this is rationing (1). This strategy is very complicated because of the difficulty in evaluating health service utilization, while it is easy to implement. Ratio can be applied to one of the four WHO’s mentioned model in its 2000 report experienced in the world (not subsidizing or investing in high-cost interventions, not subsidizing low-cost interventions, all interventions in the same proportion and no regular relationship to cost or frequency) (2). Evidently, lack of subsidizing low-cost interventions approach is the most problematic choice of policymakers (1, 2). However, any method adopted in this field should lead to improved health equity in addition to increasing efficiency and effectiveness (2).

Regarding the drug delivery system, we know that drugs sold in pharmacies are divided into two general categories, medications without a prescription (over-the-counter or OTC drugs) and medications with the doctor’s prescription (1). Over-the-counter medications are primarily used to treat symptoms such as colds, digestive disorders or other temporary pains. Research has shown that only 42% of self-medication with these drugs has been effective (3). Although there is no accurate data on the use of these drugs in Iran, that around $ 100 million annually were sold at drugstores, that is about 10% of the total sales of pharmacies (4). Over-the-counter medications have six of the top 10 best-selling drugs in the country and make about 26.5% of the medications prescribed by doctors (4).

Regarding the recent decision of the Iranian Food and Drug Administration (IFDA) to remove OTC drugs from the insurance coverage, the following is stated (5):

1- According to the Iranian Ministry of Health published list, analgesics, antacids and common colds are the majority of drugs that are on the list of 174 OTC drugs and are a part of the drug therapy for most patients (6).
2- Common Cold mostly afflicts adults and children 2–3 times and 5–6 times a year respectively and leads to the referral of patients to the doctor (7). Major drugs prescribed for these patients are on the list of OTC drugs.
3- Health insurance companies currently cover only 74.6% of the drugs prescribed by doctors, and 25.4% of them are not covered by insurance. Since 26.5% of the drugs prescribed by doctors are in the OTC drug group (4), with this decision, virtually half (51.9%) of the prescribed drugs will be excluded from insurance coverage. On the other hand, health insurance companies cover only 70% of the cost of medication prescribed by doctors, and the remaining, 30%, will be paid by patients. In this case, with the withdrawal of OTC drugs from insurance coverage, we will see that patients should actually pay about 70% (14.43%+51.9%) of their current pharmaceutical costs.

Since OTC drugs are one of the most important components of the treatment for common diseases in the community, and according to the reasons stated before, the withdrawal of these drugs from the health insurance coverage will increase the people out of pocket expenses for medicines. On the other hand, the continuation of this process will cause the patients to self-medicate because they might think that there is no need to go to a doctor when medications are not covered by health insurance companies. Obviously, the low socio-economic people are the first victims of such a policy. As it is mentioned in WHO report 2000: “The most common chronic approach to rationing care is to impose strict expenditure controls that do not try to target any specific disease’s group or broad category of interventions but simply limit budgetary obligations to affordable levels” (2). Therefore, health policy makers need evidence that is more accurate prior to implementing this decision.
